# Additive value of early-phase β-Amyloid-PET for the differential diagnosis of non-Alzheimer’s disease dementia

**DOI:** 10.1016/j.nicl.2026.103963

**Published:** 2026-02-09

**Authors:** Sebastian Eckenweber, Friederike Völter, Nicolai Franzmeier, Carla Palleis, Olivia Wagemann, Endy Weidinger, Sabrina Katzdobler, Elisabeth Wlasich, Katja Sandkühler, Guido Böning, Johannes Gnörich, Maximilian Scheifele, Florian Eckenweber, Daniel Janowitz, Carolin Kurz, Robert Perneczky, Katharina Bürger, Adrian Danek, Günter Höglinger, Johannes Levin, Matthias Brendel, Sonja Schönecker

**Affiliations:** a Department of Nuclear Medicine, Ludwig-Maximilians-Universität München, LMU Munich, Munich, Germany; b Institute for Stroke and Dementia Research (ISD), Munich, Germany; c Munich Cluster for Systems Neurology (SyNergy), Munich, Germany; d Institute of Neuroscience and Physiology and Department of Psychiatry and Neurochemistry, The Sahlgrenska Academy, University of Gothenburg, Mölndal and Gothenburg, Sweden; e Department of Neurology, Ludwig-Maximilians-Universität München, LMU Munich, Munich, Germany; f German Center for Neurodegenerative Diseases (DZNE), Munich, Germany; g Department of Psychiatry and Psychotherapy, Ludwig-Maximilians-Universität München, LMU Munich, Germany

**Keywords:** Early-phaseβ-amyloid-PET, Frontotemporal dementia, 4-repeattauopathy, Suspected non-Alzheimer pathophysiology, Data driven, Multinomial logistic regression

## Abstract

**Purpose:**

Recent studies demonstrated strong agreement between early-phase β-amyloid-PET perfusion imaging and glucose hypometabolism assessed by [^18^F]FDG-PET, indicating the potential of early-phase β-amyloid-PET as a surrogate biomarker of neuronal injury. We therefore aimed to investigate the additive value of early-phase β-amyloid-PET for the differential diagnosis of non-Alzheimer’s disease dementia syndromes in clinical routine.

**Materials and methods:**

[^18^F]florbetaben- and [^18^F]flutemetamol-PET scans (n = 379) performed between July 2013 and July 2021 were analyzed for their amyloid status and the presence of a neurodegenerative hypoperfusion pattern using visual assessment and z-score maps. In patients visually rated as amyloid-negative/neurodegeneration-positive (A-N+), the most likely diagnosis based on perfusion patterns was compared to the final clinical diagnosis, i.e. frontotemporal dementia or psychiatric disorders, suspected 4R-tauopathy, and suspected non-Alzheimer pathophysiology. Logistic regression models based on a data-driven selection of cerebral regions of hypoperfusion by principal component analysis were used to predict neurodegenerative disease and clinical diagnoses. Diagnostic accuracy was compared between visual assessment and the regression models.

**Results:**

Neurodegeneration status was correctly identified in 78.8% (119/151) of amyloid-negative patients through visual rating, compared to 67.5% (102/151) using logistic regression. Visual assessment assigned 75.3% (67/89) of A-N+ patients to the correct diagnostic category. In contrast, the regression model classified 69.7% (62/89) of patients.

**Conclusions:**

The current study demonstrates an additive value of early-phase β-amyloid-PET for the differential diagnosis of dementia syndromes. While visual assessment of early-phase β-amyloid-PET already provides substantial diagnostic accuracy, a data-driven analysis approach could aid in cases of uncertainty.

## Highlights

• Early-phase β-amyloid-PET reliably detects neurodegenerative perfusion patterns.
• The visual read identified neurodegeneration in 78.8% of amyloid-negative cases.
• 75.3% of A-/N+ cases were correctly assigned to diagnostic categories.
• Logistic regression models may aid in cases of uncertainty.

## Graphical abstract

**Figure:**
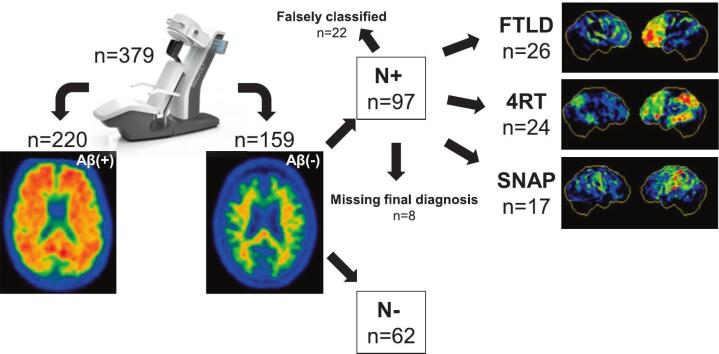

## 1 Introduction

Dementia is a neurocognitive disorder characterized by cognitive decline, that interferes with daily activities and is not explained by delirium or major psychiatric disorders (American Psychiatric Association, 2013, McKhann et al., 2011). Worldwide, approximately 55 million people live with dementia. It is the 7th leading cause of death and among the top 10 burdensome conditions among older people and has a considerable socioeconomic impact (Alzheimer's Disease International, 2015).

The majority of dementia syndromes are caused by neurodegenerative diseases, with Alzheimer’s disease (AD) being the most frequent. Other common types include frontotemporal dementia (FTD), Parkinson‘s disease dementia, or dementia due to atypical parkinsonian syndromes like the α-synucleinopathy dementia with Lewy bodies or the four-repeat-tauopathies progressive supranuclear palsy and corticobasal degeneration. While AD is pathologically characterized by an accumulation of abnormal amyloid-β (Aβ) plaques and tau neurofibrillary tangles, other neurodegenerative dementias are associated with various pathological protein aggregates like tau, TDP43 (TAR DNA-binding protein 43), FUS (RNA-binding protein fused in sarcoma) or α-synuclein. Due to clinical heterogeneity and an overlap between phenotypes and with primary psychiatric disorders, a precise prediction of the underlying pathology is difficult based on clinical features alone.

While no disease-modifying therapies have been available for treating neurodegenerative dementia syndromes so far, monoclonal antibodies targeting Aβ have recently been approved for the treatment of AD (van Dyck et al., 2023, Sims et al., 2023). In addition, for other neurodegenerative dementia syndromes like FTD or dementia due to progressive supranuclear palsy molecularly targeted therapies are currently being developed (Jiang et al., 2016, Jadhav et al., 2019, Lee et al., 2014).

Therefore, biomarkers enabling an early and accurate diagnosis are urgently needed. In recent years, various biomarkers to support the diagnosis of neurodegenerative dementia syndromes have been established. Based on the pathophysiology each measures, biomarkers used in the research of AD and other dementia syndromes can be divided according to the A/T/N classification system into biomarkers for amyloid, tau, and neurodegeneration (Jack et al., 2016). Neurodegeneration can be determined by the assessment of atrophy on structural MRI, CSF total tau, and [^18^F]fluorodeoxyglucose([^18^F]FDG)-PET. Recent studies demonstrated strong agreement between perfusion obtained by early-phase β-amyloid-PET and glucose metabolism assessed by [^18^F]FDG-PET, indicating the potential of early-phase β-amyloid-PET as a surrogate biomarker of neuronal injury (Son et al., 2020, Schmitt et al., 2021, Boccalini et al., 2022). Its ability to assess two biomarker categories simultaneously may eliminate the need for additional [^18^F]FDG-PET, thereby reducing costs, time, and radiation exposure. Furthermore, perfusion imaging has been shown to correlate with clinical severity implying its utility as an objective read-out of disease progression (Völter et al., 2025). In the current study, we therefore aimed to investigate the additive value of early phase [^18^F]florbetaben- and [^18^F]flutemetamol-PET for the differential diagnosis of amyloid-negative patients in a clinical setting. 

## 2 Material and methods

### 2.2 Study design and clinical evaluation

[^18^F]florbetaben- and [^18^F]flutemetamol-PET scans performed in a clinical routine setting at the Department of Nuclear Medicine, Ludwig-Maximilians-Universität München, Munich, Germany between July 2013 and July 2021, were analyzed to determine the amyloid status. Patients were examined at the Department of Neurology, the Institute for Stroke and Dementia Research, and the Department of Psychiatry and Psychotherapy. The cohort included cognitively unimpaired individuals (subjective cognitive impairment) and cognitively impaired patients. At the time of the β-amyloid-PET examination the suspected clinical diagnoses were mild cognitive impairment, AD dementia, behavioural variant frontotemporal dementia or primary progressive aphasia, corticobasal syndrome or primary psychiatric disorders. β-amyloid-PET was performed to confirm or exclude AD pathology in these conditions.

Scans were evaluated by a resident and an attending, i.e. an experienced nuclear medicine physician, with the final decision on amyloid positivity/negativity made by the attending. Patients whose late-phase (90–110 min p.i.) images were rated as amyloid-negative (A-) on visual inspection were considered as the primary sample. Patients were then classified as neurodegeneration-positive (A-N+) or neurodegeneration-negative (A-N-) on visual inspection of early-phase images (0–10 min p.i.). Surface projections of hypoperfusion patterns were used to support visual reading of early-phase images. A-N+ early-phase images were additionally categorized into 3 perfusion patterns: a) frontotemporal hypoperfusion, potentially compatible with FTD or severe primary psychiatric disorders, b) central hypoperfusion with additional asymmetric hypoperfusion in subcortical areas matching four-repeat tauopathies (4R-tauopathy), and c) parietooccipital hypoperfusion compatible with suspected non-Alzheimer pathophysiology (SNAP) including diagnoses like dementia with Lewy bodies or vascular dementia. Visual readers were blinded against clinical information.

The perfusion patterns were then compared to the final clinical diagnosis. A- patients were categorized according to the final clinical diagnosis into non-neurodegenerative and neurodegenerative diseases. Patients whose β-amyloid-PET was classified as A-N+ were assigned into the following clinical diagnostic categories: FTD or severe psychiatric disorder, suspected 4R-tauopathy, and SNAP. Diagnosis was made according to current diagnostic criteria (Rascovsky et al., 2011, Gorno-Tempini et al., 2011, Hoglinger et al., 2017, Armstrong et al., 2013, Jack et al., 2016, American Psychiatric Association, 2022). It is important to note that the A-N+ group, while classified as N+, also included some patients with non-neurodegenerative conditions. This is particularly evident in the FTD or severe psychiatric disorder and the SNAP category including patients with primary psychiatric disorders or vascular dementia respectively. Given that visual assessment of neurodegeneration status has limited specificity and sensitivity patients may exhibit perfusion abnormalities typically associated with neurodegeneration but are later diagnosed with non-neurodegenerative disorders. This diagnostic limitation is reflected in our classification system, where certain clinical conditions, like severe primary psychiatric disorders, are grouped with neurodegenerative diseases due to overlapping symptoms and shared imaging features. As all patients in the study were amyloid-negative in the late phase, AD was not grouped.

To test whether data driven approaches can enhance diagnostic accuracy of early-phase β-amyloid-PET a binary logistic regression model predicting the presence of a neurodegenerative disease as well as a multinomial logistic regression model predicting ternary clinical diagnoses were calculated based on a data-driven selection of cerebral regions of hypoperfusion. Measures of diagnostic accuracy were compared between the visual read and the logistic regression models.

In patients, a clinical neurological examination as well as neuropsychological testing consisting of the Mini-Mental-State-Examination (MMSE) and optionally CERAD plus battery including Trail Making Test A and B as well as verbal fluency tests were performed. Age, education, and disease duration, i.e. subjective symptom onset to performance of β-amyloid-PET, as well as laboratory parameters for metabolic causes of dementia (vitamin B12, thiamine, folate levels, thyroid and liver function) were assessed. MRI was performed on 1.5 T and 3.0 T scanners, using at least a T1w sequence for atrophy assessment and a T2w-Flair sequence for screening of leukoencephalopathy. Cerebrospinal fluid was collected for the assessment of phosphorylated tau (threshold: p-tau < 61 pg/ml), total tau (threshold: <445 pg/ml), and Aβ42/40 (threshold < 5.5%) and Aβ concentration (threshold: >375 pg/ml). Demographics of of the study sample are provided in Table 1.

**Table 1:** Demographics of the study sample.

	Non-neurodegenerative	Neurodegenerative	*p*-value	FTD or severe Psychiatric Disorder	Suspected 4R-tauopathy	SNAP	*p*-value
	N = 56	N = 95		N = 32	N = 32	N = 25	
Sex (f/m)	25/31	51/44	0.29	18/14	16/16	11/14	0.65
Age	68.2 ± 11.4	69.3 ± 8.5	0.94	65.8 ± 10.4^c^	69.1 ± 8.1	73.2 ± 6.3^a^	**0.01**
Education	14.2 ± 3.1	13.2 ± 3.8	**0.03**	14.5 ± 4.4	13.0 ± 4.2	14.1 ± 3.0	0.07
MMSE	26.8 ± 3.4	25.0 ± 4.3	**0.01**	24.8 ± 4.7	24.7 ± 4.5	26.1 ± 2.6	0.69
Disease Duration (mo)	38.7 ± 40.5	36.6 ± 27.3	0.53	46.6 ± 46.6	44.8 ± 22.8	38.2 ± 36.3	0.17
Follow-Up Duration (mo)	17.1 ± 18.4	14.2 ± 17.2	0.40	11.0 ± 13.8	21.3 ± 21.1^c^	8.4 ± 14.2^b^	**0.02**

Significantly different compared to ^a^FTD or severe Psychiatric Disorder, ^b^suspected 4R-tauopathy, ^c^SNAP *f* female, *FTD* frontotemporal dementia, m male, *MMSE* Mini-Mental State Examination, *mo* months, *4R-tauopathy* 4-repeat-tauopathy, *SNAP* suspected non-Alzheimer pathophysiology, *y* years Classification of A- patients into neurodegenerative and non-neurodegenerative diseases was derived from the final clinical diagnosis. A-N + patients were further assigned to the categories FTD (Rascovsky et al., 2011, Gorno-Tempini et al., 2011) or severe psychiatric disorder (American Psychiatric Association, 2022), suspected 4R-tauopathy (Hoglinger et al., 2017, Armstrong et al., 2013), and SNAP (Jack et al., 2016) according to current diagnostic criteria.							

### 2.3 Radiosynthesis and PET imaging

[^18^F]flutemetamol was synthesized in house on a FASTlab synthesizer model by the reaction of N-[4(6-ethoxymethoxybenzothiazol-2-yl)-2-nitro-phenyl]-N-methylformamide (AH111907) with [^18^F]fluoride and purified by solid phase extraction followed by formulation into [^18^F]flutemetamol injection (Senda et al., 2015). [^18^F]florbetaben was purchased commercially. [^18^F]flutemetamol-PET (n = 255) was performed with dual phase 0–10 min (early-phase) and 90–110 min (late-phase) emission recordings after administration of 187 +/- 10 MBq [^18^F]flutemetamol. [^18^F]florbetaben (n = 124) followed the same protocol after the intravenous injection of 300.9 +/- 16.8 MBq. Low-dose CT scans preceded the early- and late-phase acquisition (Schmitt et al., 2021).

Scans were performed on a Siemens Biograph 64 True X (Siemens, Erlangen, Germany), a Siemens mCT (Siemens, Erlangen, Germany) or a GE Discovery 690 PET/CT (GE Healthcare). Alignment to the Siemens Biograph was achieved using harmonized brain PET reconstruction protocols based on Hoffman phantom measurements (Brendel et al., 2020).

### 2.4 Early-phase amyloid-PET analysis

Stereotactical normalization of all PET scans was performed in HERMES Gold (V4.17; HERMES medical solutions AD, Stockholm, Sweden). Regional standardized uptake value ratios (SUVr) and SUVr z-scores of the included AAL atlas (modified version with 47 regions) were calculated using the cerebellar gray matter as reference. Three-dimensional stereotactic surface projections (3D-SSP) (Minoshima et al., 1995) were created with Neurostat (Department of Radiology, University of Washington, Seattle, WA, USA) for visual image interpretation. Neurostat compared individual early-phase tracer uptake to historical [^18^F]FDG-PET images from a healthy age-matched cohort (n = 18). Visual assessment of axial slices and 3D-SSP images was carried out by an expert in Nuclear Medicine (M.B.) using tracer uptake and z-score maps (global mean scaling). Early-phase images were classified as either A-N+ or A-N-. In A- patients the presence of a neurodegenerative perfusion pattern was compared to the binary clinical diagnosis, i.e. non-neurodegenerative vs. neurodegenerative disease. In contrast, in A-N+ patients perfusion patterns were compared to the ternary clinical diagnostic categories, i.e. FTD or severe psychiatric disorder, suspected 4R-tauopathy, and SNAP.

### 2.5 Statistical analysis

Data were analyzed using IBM SPSS Statistics (Version 28.0 Armonk, NX: IBM Corp.). Demographic and clinical data were compared between patient groups, i.e. non-neurodegenerative and neurodegenerative patients as well as the diagnostic categories FTD or severe psychiatric disorders, suspected 4R-tauopathy and SNAP according to the final clinical diagnosis of A-N+ patients, by *post hoc* Bonferroni corrected ANOVA or Kruskal Wallis test, as appropriate. Chi-square tests evaluated sex differences. Standard statistical significance was set at *p* < 0.05. Diagnostic accuracy of the visual read for predicting neurodegeneration status and final clinical diagnoses of A-N+ patients was evaluated by receiver operating characteristic (ROC) curves and the respective area under the curve (AUC), with confidence intervals derived via the bootstrap method.

We tested whether a data driven approach can enhance diagnostic accuracy of early-phase β-amyloid-PET. Given the exploratory aim of identifying latent regional patterns rather than supervised classification, principal component analysis (PCA) was preferred over alternative multivariate methods. To identify groups of similar brain regions based on regional SUVr z-scores of early-phase images of A-N+ patients, our set of 47 cerebral regions was subjected to a PCA with direct oblimin rotation. Following the advice of Stevens (Stevens, 2009), variables with factor loadings below 0.4, which are considered to contribute minimally to the component structure, were eliminated from the analysis and the PCA run anew. Components consisting of two or fewer variables with substantial loadings (≥ 0.4) and explaining less than 10% of the total variance were excluded a priori, as such components provide limited structural information and are considered unstable in exploratory PCA. Components were labeled *post hoc* according to the pattern of cerebral regions. For further analysis, PCA-based component scores were calculated from the factor loading weighted variables of each component. Component scores of A-N+ patients were compared via Kruskal-Wallis and *post-hoc* Bonferroni corrected Mann-Whitney tests between clinical diagnostic categories. Using PCA-based component scores we calculated a binary logistic regression model with backward selection for predicting neurodegeneration status and a multinomial logistic regression model with backward selection for predicting final clinical diagnoses of A-N+ patients. Measures of diagnostic accuracy were compared between visual assessment of early-phase β-amyloid-PET images and the calculated regression models.

To address potential overfitting, we additionally applied a more conservative approach and performed a repeated 10-fold cross-validation including PCA and logistic regression (50 repetitions). Only variables with factor loadings ≥ 0.4 were retained, and PCA components with eigenvalues > 1 were used as predictors. Backward selection was applied within each training fold.

To evaluate whether findings are tracer specific, performance of the visual read and the lostic regression models were compared between tracers. AUCs were compared using DeLong’s test for independent ROC curves.

In a subset of 15 A-N+ patients who underwent both early-phase β-amyloid-PET and [^18^F]FDG-PET within less than three months, SUVr z-scores were compared. For each region, z-scores were averaged across patients to obtain regional mean values for both modalities. Normality of the regional mean values was assessed using the Shapiro-Wilk test. As early-phase β-amyloid-PET data were not normally distributed, Spearmans’s rank correlation coefficient (ρ) was used as the primary measure of association, complemented by Pearson’s correlation coefficient (r).

### 2.6 Data availability

The data that support the findings of this study are available from the corresponding author upon reasonable request and submission of a formal project outline. Data are not publicly available due to privacy restrictions.

## 3 Results

### 3.1 Patient characteristics

No adverse events were observed related to the PET scans. 159/379 β-amyloid-PET scans were rated as A- (42%) and thereof 62 (39%) as A-N- and 97 as A-N+ (61%). 8 A-N+ patients had to be excluded due to missing information regarding the final clinical diagnosis. Of the remaining 89 A-N+ patients, 39 had frontotemporal hypoperfusion, 25 central hypoperfusion with additional asymmetric hypoperfusion in subcortical areas, and 25 parietooccipital hypoperfusion (Table 1, Table 2, Table 3, Supplementary Tab. 1 and Supplementary Fig. 1).

**Table 2:** Contingency tables for determining neurodegeneration status.

According to visual assessment of axial slices and 3D-SSP images				According to the binary logistic regression model			
	Final clinical diagnosis				Final clinical diagnosis		
Predicted diagnosis	Non-neurodegenerative	Neurodegenerative	Total	Predicted diagnosis	Non-neurodegenerative	Neurodegenerative	Total
N-	43	19	62	N-	43	36	79
N+	13	76	89	N+	13	59	72
Total	56	95	151	Total	56	95	151

Final clinical diagnosis, used as the reference standard for classification into non-neurodegenerative and neurodegenerative diseases, was established according to consensus diagnostic criteria (Rascovsky et al., 2011, Gorno-Tempini et al., 2011, Hoglinger et al., 2017, Armstrong et al., 2013, Jack et al., 2016, American Psychiatric Association, 2022). N- indicated prediction of non-neurodegenerative diseases, while N + indicates prediction of neurodegenerative diseases based on either visual assessment of early-phase β-amyloid-PET images or a binary logistic regression model using mean component scores of components #6, #7 and #8.

**Table 3:** Contingency tables for determining final clinical diagnosis.

According to visual assessment of axial slices and 3D-SSP images					According to the multinomial logistic regression model				
	Final clinical diagnosis			Total		Final clinical diagnosis			Total
Perfusion pattern	FTD or severe psychiatric disorder	Suspected 4R-tauopathy	SNAP		Predicted diagnosis	FTD or severe psychiatric disorder	Suspected 4R-tauopathy	SNAP	
Frontotemporal hypoperfusion	26	6	7	39	FTD or severe psychiatric Disorder	26	6	7	39
Central hypoperfusion	0	24	1	25	Suspected 4R-tauopathy	4	23	5	32
Parietooccipital hypoperfusion	6	2	17	25	SNAP	2	3	13	28
Total	32	32	25	89	Total	32	32	25	89

*FTD* frontotemporal dementia, *4R-tauopathy* 4-repeat-tauopathy, *SNAP* suspected non-Alzheimer patholophysiology Final clinical diagnosis, used as the reference standard for classification into FTD (Rascovsky et al., 2011, Gorno-Tempini et al., 2011) or severe psychiatric disorder (American Psychiatric Association, 2022), suspected 4R-tauopathy (Hoglinger et al., 2017, Armstrong et al., 2013), and SNAP (Jack et al., 2016) was established according to consensus diagnostic criteria. Predicted diagnoses were determined based on either visual assessment of early-phase β-amyloid-PET images or a multinomial logistic regression model using mean component scores of components #2, # 3, #4, #5, and #6.									

According to the final clinical diagnosis 95/151 (62.9%) patients were classified as having a neurodegenerative disease, while 56/151 (37.1%) were not classfied as neurodegenerative. Neurodegenerative patients had significantly lower MMSE scores, and lower education levels compared to non-neurodegenerative patients. Groups did not differ in terms of sex, age, disease or follow-up duration.

Out of 89 patients visually rated as A-N+ 32 were diagnosed with FTD or severe psychiatric disorder, 32 with suspected 4R-tauopathy and 25 with SNAP. SNAP patients were significantly older than FTD or severe psychiatric disorder patients and had a shorter follow-up duration compared to suspected 4R-tauopathy patients. No significant group differences were observed in sex, education, disease duration, and MMSE (Table 1 and Supplementary Tab. 1).

### 3.2 Diagnostic accuracy based on the visual assessment of early-phase perfusion amyloid-PET imaging

**Fig. 1:**
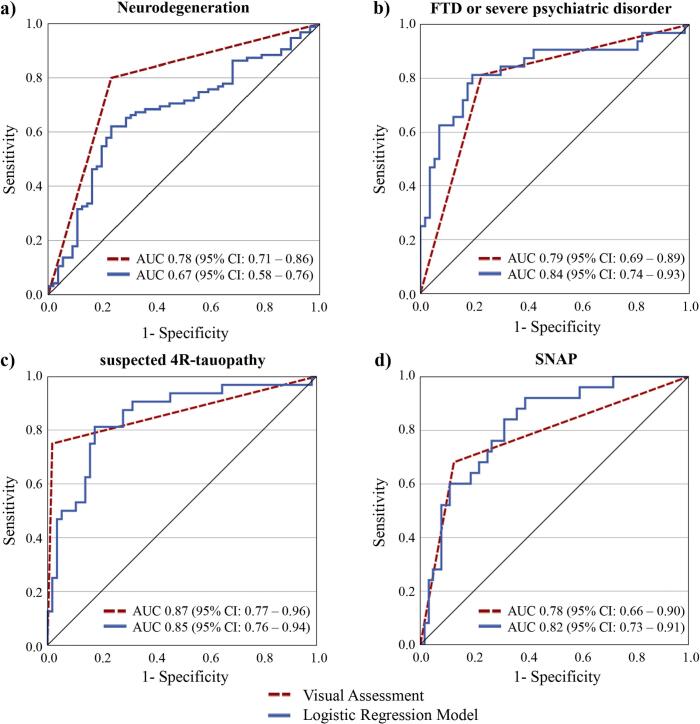
Receiver operating characteristic curves for the visual neurodegeneration assessment of early-phase β-amyloid-PET images versus logistic regression models. (a) prediction of neurodegeneration status across all patients rated as A-, (b-d) prediction of the final clinical diagnosis, i.e. FTD or severe psychiatric disorder (b), suspected 4R-tauopathy (c) and SNAP (d). The black diagonal line is the ROC curve reference line. 95% CI, 95% confidence interval; AUC, area under the curve.

Based on visual assessment of early-phase images, an expert in Nuclear Medicine accurately assessed neurodegeneration status in 119/151 (78.8%) patients (Table 2). 43/62 (69.4%) patients rated as A-N- were correctly classified, most of whom had a final clinical diagnosis of mild (n = 13) and subjective cognitive impairment (n = 11) without evidence of neurodegenerative disease respectively or primary psychiatric disorder (major depressive disorder n = 4, alcohol use disorder n = 1, schizophrenia n = 1, somatic symptom disorder n = 1). Most patients who were incorrectly categorized as A-N- (n = 19; 30.6%) had a final clinical diagnosis of suspected 4R-tauopathy (n = 9). While 76/89 (85.4%) patients rated as A-N+ were correctly classified, 13 (14.6%) were falsely categorized as A-N+, most of whom had a final diagnosis of primary psychiatric disorder (major depressive disorder n = 3, adjustment disorder n = 3, schizophrenia n = 1, alcohol use disorder n = 1) or vascular dementia (n = 4). Diagnostic accuracy for determining neurodegeneration status measured via the AUC was 0.78 (95% Confidence Interval: 0.71 – 0.86), indicating good discriminatory ability (Šimundić, 2009). The sensitivity was 80.0%, while the specificity was 76.8% (Fig. 1).

[^18^F]flutemetamol, neurodegeneration status was correctly classified in 83/108 (76.9%), while for [^18^F]florbetaben, correct classification was achieved in 36/43 (86.0%) paitents. The discriminative performance of the visual read was comparable between tracers, with AUCs of 0.77 (95% Confidence Interval: 0.67–0.86) and 0.82 (95% Confidence Interval: 0.68 – 0.97) for [^18^F]flutemetamol and [^18^F]florbetaben, respectively (*p* = 0.47, DeLong’s test for independent ROC curves, Supplementary Fig. 2) indicating no statistically significant difference.

**Fig. 2:**
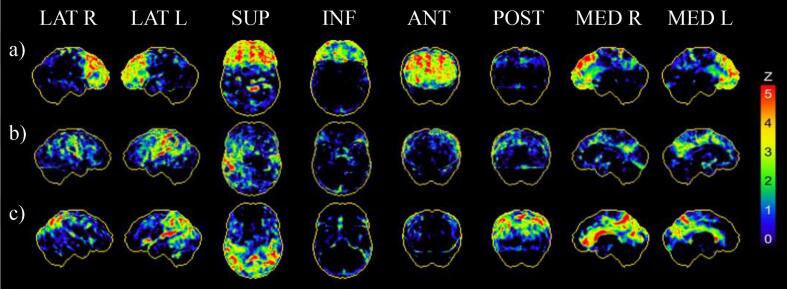
Representative 3D-SSP images (z-score maps) of early-phase [^18^F]flutemetamol and [^18^F]florbetaben-PET for the three different clinical diagnoses of: (a) FTD or severe psychiatric disorder demonstrating frontotemporal hypoperfusion. (b) suspected 4-repeat-tauopathy demonstrating central hypoperfusion and additional asymmetric subcortical hypoperfusion. (c) suspected non-Alzheimer pathophysiology demonstrating parietooccipital hypoperfusion. Surface projections from R. right; L. left; LAT lateral; SUP. superior; INF. inferior; ANT. anterior; POST. posterior; MED. Medial.

Patients classified as A-N+ were categorized into FTD or severe psychiatric disorder, suspected 4R-tauopathy, and SNAP according to the final clinical diagnosis. The expert in Nuclear Medicine assigned 67/89 (75.3%) patients to the correct diagnostic category (Tab. 3). 13 patients were falsely rated as FTD or severe psychiatric disorder, 1 patient as suspected 4R-tauopathy and 8 patients as SNAP. The AUC for discriminating 4R-tauopathies from the other disease entities was highest at 0.87 (95% Confidence Interval: 0.77–0.96, sensitivity 75.0%, specificity 98.2%), followed by the AUC for discriminating FTD or severe psychiatric disorder at 0.79 (95% Confidence Interval: 0.69–0.89, sensitivity 81.3% specificity 77.2%) and the AUC for discriminating SNAP at 0.78 (95% Confidence Interval: 0.66–0.90, sensitivity 68.0%, specificity 87.5%; Fig. 1). Representative 3D-SSP images are provided in Fig. 2.

In a tracer-stratified analysis, the diagnostic accuracy of the visual read was comparable between tracers. Using deLong’s test, no significant differences in AUCs were observed between tracers, indicating that the visual read performance was not tracer-specific (Supplementary Fig. 3).

### 3.3 Principal component analysis

We furthermore tested whether regression models based on a data-driven selection of cerebral regions of hypoperfusion can enhance diagnostic accuracy of early-phase β-amyloid-PET. PCA with direct oblimin rotation identified 10 components with eigenvalues above 1 (Supplementary Fig. 4).

**Fig. 3:**
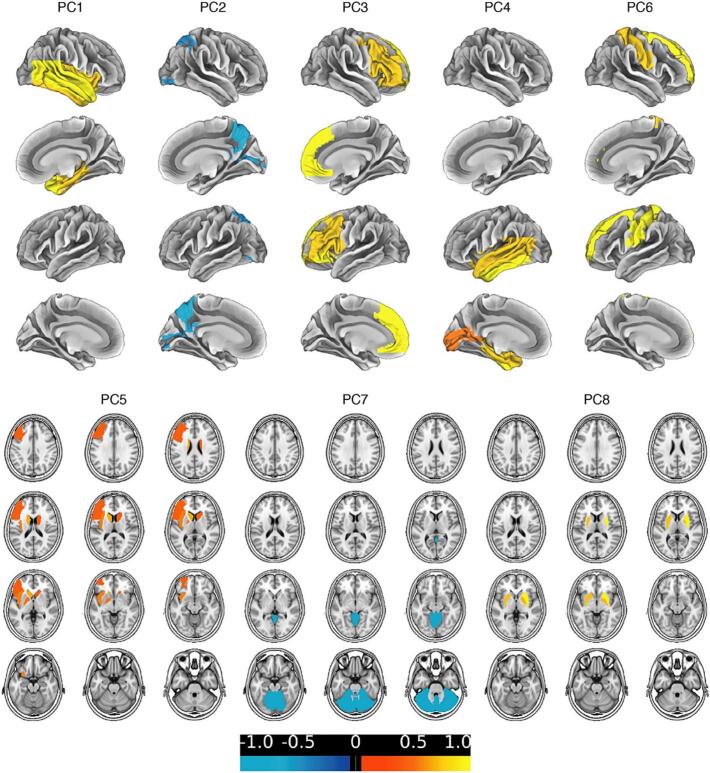
Principal components of early-phase [^18^F]florbetaben- and [^18^F]flutemetamol SUVr z-scores. Rotated regional weights of early-phase [^18^F]florbetaben and [^18^F]flutemetamol SUVr z-score components identified by applying principal component analysis on 47 cerebral regions. The colors represent each component's region-specific weights (range from −1.0 to 1.0).

Components containing only two or less high-loading variables were excluded from the analysis, resulting in an 8-component solution which explained 76.3% of variance. The item left inferior parietal cortex was excluded due to factor loadings below 0.4. The resulting components comprised the following regions (Fig. 3 and Supplementary Tab. 2):

• component #1 = Right temporal lobe (variance explained 27.3%)
• component #2 = Parietooccipital junction (variance explained 13.8%)
• component #3 = Frontal lobe (variance explained 9.4%)
• component #4 = Left Temporal lobe (variance explained 7.4%)
• component #5 = Caudate nucleus and thalamus (variance explained 6.9%)
• component #6 = Pre- and postcentral cortex (variance explained 4.6%)
• component #7 = Cerebellum (variance explained 4.0%)
• component #8 = Pallidum and putamen (variance explained 3.0%)

### 3.4 Comparison of component scores between groups

**Fig. 4:**
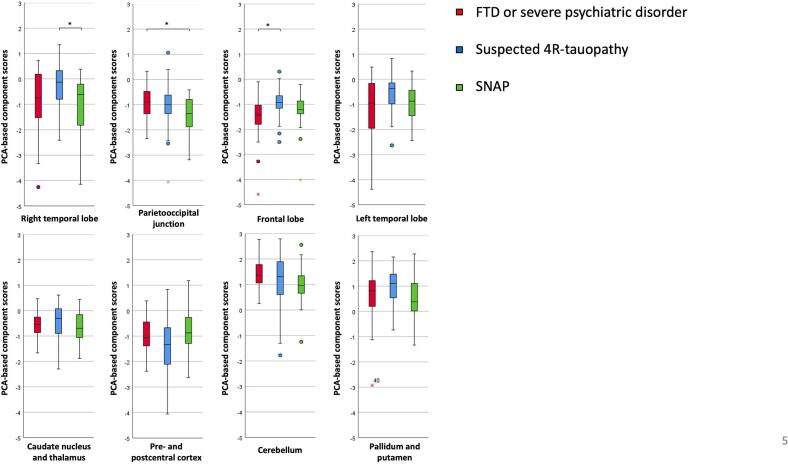
Comparison of component scores between groups. Component scores were calculated from the factor loading weighted variables of each of the components identified by applying principal component analysis on 47 cerebral regions. * indicates significant differences.

When comparing component scores between the three clinical diagnostic categories of A-N + patients, Kruskal-Wallis test detected significant group differences in component #1 (right temporal lobe), #2 (parietooccipital junction), and #3 (frontal lobe) (Fig. 4). While in the right temporal lobe, perfusion was significantly lower in SNAP compared to suspected 4R-tauopathy patients, in the parietooccipital junction perfusion in SNAP was lower compared to FTD or severe psychiatric disorder patients. In the frontal lobe in FTD or severe psychiatric disorder patients’ perfusion was significantly lower compared to suspected 4R-tauopathy patients. No significant group differences regarding the other components could be detected. However, FTD or severe psychiatric disorder and SNAP patients additionally displayed comparatively low perfusion in the left temporal lobe. While suspected 4R-tauopathy patients demonstrated lowest perfusion in the pre- and postcentral cortex, they had slightly higher perfusion in the caudate nucleus and thalamus compared to the other groups. For all diagnostic categories a relative hyperperfusion in the cerebellum as well as the pallidum and putamen was observed.

Component scores of the eight A-N+ patients excluded due to missing final clinical diagnosis did not differ significantly from those of the evaluated diagnostic categories (Supplementary Fig. 5).

### 3.5 Diagnostic accuracy based on logistic regression models

The optimal binary logistic regression model for predicting neurodegeneration status (*p* = 0.01, *R*^2^ = 0.099) using mean component scores of components #6, #7 and #8 (pre- and postcentral cortex, cerebellum and pallidum and putamen) classified 102/151 (67.5%) patients correctly and resulted in an AUC of 0.67 (95% Confidence Interval: 0.58 – 0.76, sensitivity 62.1%, specificity 76.8%), corresponding to sufficient discriminatory ability (Fig. 1, Tab. 2). A more conservative approach, performing repeated cross-validation including PCA and logistic regression, still yielded a mean classification accuracy of 59.1% and a mean AUC of 0.545, indicating a reduced but non-random predictive performance under stringent validation.

When comparing the diagnostic accuracy based on regression models for patients scanned with either [^18^F]flutemetamol or [^18^F]florbetaben, the model for [^18^F]flutemetamol correctly classified 70/108 (64.8%), resulting in an AUC of 0.65 (95% Confidence Interval: 0.54 – 0.76, sensitivity 66,7%, specificity 66.7%). The model for [^18^F]florbetaben correctly classified 32/43 patients (74.4%), with an AUC of 0.74 (95% Confidence Interval: 0.58 – 0.90, sensitivity 65.5%, specificity 92.9%). Comparison of AUCs using DeLong’s test for independent ROC curves showed no statistically significant difference between the models (*p* = 0.30), indicating comparable discriminatory performance (Supplementary Fig. 2).

The optimal multinomial logistic regression model for predicting the final clinical diagnosis in A-N + patients using component scores of components #2, #3, #4, #5, and #6, (parietooccipital junction, frontal lobe, left temporal lobe, caudate nucleus and thalamus and pre-and postcentral cortex) as predictor variables classified 62/89 (69.7%) patients correctly (Tab. 3). Component selection was based on a backward stepwise selection procedure within the multinomial logistic regression framework rather than on univariate group comparisons, which explains why the final model includes components beyond thosed identified by the Kruskal-Wallis test. Component #1 did not provide additional independent predictive value in the multivariable model and was therefore excluded during backward selection. The AUC for discriminating suspected 4R-tauopathies from the other disease entities was highest at 0.85 (95% Confidence Interval: 0.76 – 0.94, sensitivity 81.3%, specificity 82.5%), followed by the AUC for discriminating FTD or severe psychiatric disorder at 0.84 (95% Confidence Interval: 0.74 – 0.93, sensitivity 81.3%, specificity 80.7%) and the AUC for discriminating SNAP at 0.82 (95% Confidence Interval: 0.73 – 0.91, sensitivity 92.0%, specificity 60.9%)(Fig. 1). Regression coefficients of both models are summarized in Supplementary Fig. 6 and 7.

A repeated 10-fold cross-validation with PCA and multinomial logistic regression resulted in a mean classification accuracy of 50.0% ± 17.25%. Despite the reduced overall accuracy, class-wise discrimination remained moderate and consistent across folds, with mean AUC of 0.745 ± 0.174 for FTD or severe psychiatric disorders, 0.731 ± 0.165 for suspected 4R-tauopathies, and 0.734 ± 0.170 for SNAP, confirming the reliability of the model in a more conservative framework.

A supplemental tracer-stratified analalysis showed comparable diagnostic performance for the logistic regression models between [^18^F]flutemetamol and [^18^F]florbetaben-PET. While model performance was numerically slightly higher for [^18^F]florbetaben-PET, DeLong’s test revealed no statistically significant differences in AUCs between tracers, indicating that the findings were not tracer-specific (Supplementary Fig. 3).

### 3.6 Comparison of diagnostic accuracy based on visual assessment of early-phase images and calculated logistic regression models

Visual assessment of early-phase images for predicting neurodegeneration status outperformed the binary logistic regression model in terms of diagnostic accuracy, only the specificity was identical (76.8%). In patients visually rated as A-N-, the regression model correctly identified 12/19 falsely rated images as neurodegenerative but misclassified 8 scans as neurodegenerative despite actually being non-neurodegenerative. In patients visually rated as N+, the model reclassified 8/13 falsely rated images correctly as non-neurodegenerative, whereas it classified 29 scans as N- despite them actually being neurodegenerative.

Regarding the prediction of the final clinical diagnosis in patients rated as A-N+, visual assessment of early-phase images performed better compared to the multinomial logistic regression model in terms of AUC regarding the discrimination of suspected 4R-tauopathy from the other disease entities. However, the regression model performed better regarding the discrimination of FTD or severe psychiatric disorder and SNAP patients respectively from the other entities. There were 13 patients in whom the expert in Nuclear Medicine made the correct diagnosis while the prediction model assumed an incorrect one. In contrast, there were 8 patients in whom the model correctly predicted the final clinical diagnosis whereas the expert in Nuclear Medicine was wrong.

### 3.7 Spatial coupling between early-phase β-amyloid-PET and FDG-PET

Across the 47 cerebral regions, early-phase β-amyloid-PET and [^18^F]FDG-PET showed strong spatial coupling in the investigated 15 A-N + patients. Spearman’s correlation was ρ = 0.733, and Pearson’s correlation was r = 0.876, corresponding to non-shared variance of 46% (Spearman) and 38% (Pearson). The non-normal distribution of early-phase β-amyloid-PET values supports Spearman’s ρ as the more robust measure.

## 4 Discussion

We present the first study demonstrating an additive value of early-phase β-amyloid PET for the differential diagnosis of amyloid-negative patients. Through visual assessment of axial slices and 3D-SSP images, we were able to predict the presence of a neurodegenerative disease, as well as in patients visually classified as N+, the final clinical diagnosis with high accuracy. A data-driven approach may additionally be beneficial in ambiguous cases.

The A/T/N classification system, widely established in AD research and diagnostics, may also be beneficial in the diagnostic workup of non-AD neurodegenerative disorders. While β-amyloid-PET is recognized as a valid marker of Aβ deposition (Jack et al., 2016), its early-phase has only recently been proposed as a surrogate of neuronal injury (Son et al., 2020, Schmitt et al., 2021). Neuronal injury has traditionally been assessed by structural MRI, CSF total tau, or FDG-PET. Recent studies, however, demonstrated strong agreement between perfusion deficits obtained by early-phase β-amyloid-PET and glucose metabolism assessed by [^18^F]FDG-PET (Schmitt et al., 2021). In line with these data, in our study we were able to correctly determine neurodegeneration status in 78.8% of patients based on visual assessment of early-phase β-amyloid-PET.

69.4% of patients rated as A-N- were correctly classified as N-. Previous studies have shown high negative predictive values of [^18^F]FDG-PET for the detection of neurodegenerative processes, with less than 10% of patients with a preserved [^18^F]FDG-PET scan progressing to degenerative dementia over several years (Nobili et al., 2018, Silverman et al., 2001). However, these studies included amyloid-positive patients undergoing an AD process. The lower negative predictive value for the detection of neurodegeneration in our study is probably due to the preselection of solely amyloid-negative patients and the nonetheless heterogeneous patient cohort with varying degrees and stages of cognitive deficits. Furthermore, clinical diagnostic certainty in the patient group falsely classified as N- was limited, with most patients being classified as “possible” or “suspected” neurodegenerative disease, making a clinical misdiagnosis possible.

β-amyloid- and [^18^F]FDG-PET have been shown to provide valuable and complementary information, for the diagnostic work-up of neurodegenerative diseases. Consequently, diagnostic algorithms have been proposed that vary the order of amyloid- and [^18^F]FDG-PET assessments depending on the most likely suspected diagnosis (Chételat et al., 2020, Frisoni et al., 2024). After an amyloid-negative PET scan, an additional [^18^F]FDG-PET can address further differential diagnoses, particularly within the FTD spectrum. Our data suggest that early-phase β-amyloid-PET is also suited to capture neurodegenerative patterns and thus could represent an appropriate alternative to [^18^F]FDG-PET. The ability of β-amyloid-PET to assess two biomarker categories in a one-stop-shop examination may therefore eliminate the need for an additional [^18^F]FDG-PET and thereby reduce costs, time, and radiation exposure.

The treatment of non-AD neurodegenerative diseases has so far been limited to symptomatic therapies and aspects of patient care. However, disease-modifying drug trials are underway for diverse neurodegenerative diseases like frontotemporal dementia (Jiang et al., 2016, Jadhav et al., 2019, Lee et al., 2014) and 4R-tauopathies (VandeVrede et al., 2020, Cummings et al., 2023). In light of these trials, biomarkers that enable an early and accurate diagnosis become increasingly important. Furthermore, high diagnostic accuracy is essential for estimating prognosis and optimizing patient care. In our study, we were able to accurately assign 75.3% of A-N+ patients based on visual assessment of early-phase β-amyloid-PET images to the final clinical diagnostic category. Previous studies demonstrated a similar diagnostic accuracy of [^18^F]FDG-PET in distinguishing neurodegenerative disorders (Foster et al., 2007, Houssein et al., 2023, Lesman-Segev et al., 2021), which highlights the potential of early-phase β-amyloid-PET as a surrogate of neuronal injury. In contrast, a multinomial logistic regression model based on a data-driven selection of cerebral regions achieved lower classification accuracy of 69.7%. However, the logistic regression model performed slightly better in discriminating FTD or severe psychiatric disorder and SNAP patients from the other disease entities, with higher specificity for detecting FTD or severe psychiatric disorder and higher sensitivity for detecting SNAP patients. A more conservative approach using repeated cross-validation, applied to address potential overfitting, yielded an even lower accuracy, indicating reduced but non-random predictive performance. Despite this, class-wise discrimination remained moderate and consistent, with AUCs of 0.73 – 0.75 across the main diagnostic categories. Therefore, the model could provide complementary information, especially in cases where visual inspection remains inconclusive, and could be used to verify or reassess diagnoses when discrepancies arise between visual assessment and the regression model. Furthermore, the regression model could be applied depending on whether the objective is to prioritize sensitivity or specificity. Future studies in independent cohorts are required to validate these models and determine their potential utility in clinical practice.

As the name implies, patients with a final clinical diagnosis of FTD or severe psychiatric disorder demonstrated low perfusion in the frontal and temporal lobes (Antonioni et al., 2023). SNAP patients showed low perfusion in the temporal lobes as well and additionally demonstrated lowest perfusion in the parietooccipital junction, a region that, among others, consists of the precuneus and posterior cingulate cortex and that is typically affected by neurodegeneration in AD (DeTure and Dickson, 2019). While milder, the pattern of neurodegeneration in SNAP is known to be similar in distribution to that observed in AD (Chiaravalloti et al., 2019). In agreement with previous studies showing an early affection of the motor cortex in 4R-tauopathies (Kovacs et al., 2020, Albrecht et al., 2017), in our study patients with suspected 4R-tauopathy demonstrated lowest perfusion in the pre- and postcentral cortex. Interestingly, however, suspected 4R-tauopathy patients showed slightly higher perfusion in the caudate nucleus and thalamus compared to the other groups. However, neuronal injury of the caudate nucleus and thalamus is not specific for 4R-tauopathies but can also occur in other disorders, like FTD (Schonecker et al., 2018, McKenna et al., 2023), or AD (Power and Looi, 2015). For all three disease entities, we detected a relative hyperperfusion in the cerebellum as well as the pallidum and putamen. When evaluating PET images in the context of neurodegenerative diseases, the emphasis is usually on assessing hypometabolism or hypoperfusion. In agreement with our data, however, hyperperfusion and hypermetabolism of the cerebellum, pallidum, and putamen have consistently been described in neurodegenerative disorders (Katzdobler et al., 2023, Vella et al., 2014, Blum et al., 2018) and have been interpreted as compensatory neuronal activation. Importantly, the cerebellar finding was derived from a PCA-based component comprising bilateral cerebellar regions and the vermis and does not correspond to, nor fully overlap with, the cerebellar gray matter reference region used for SUVr normalization. Therefore, our data do not allow conclusions regarding the suitability or validity of cerebellar gray matter as a reference region. Morover, cerebellar perfusion did not differ significantly between disease entities, indicating that this relative hyperperfusion does not affect the comparative interpretation of our results. As demonstrated, perfusion measured by early-phase β-amyloid-PET in clinically diagnosed patients mirrors the established pattern of neurodegeneration of these disease entities. Early-phase β-amyloid-PET could therefore provide a valuable tool for the differential diagnosis of neurodegenerative diseases.

Interestingly, the cerebral regions used to visually categorize early-phase images into three perfusion patterns are in good agreement with those identified by the regression model for differentiating neurodegenerative diseases. This consistency assures us aobout the validity of both methods. Therefore, when assessing early-phase β-amyloid-PET for diagnostic purposes, priority should be given to evaluating perfusion in these key regions, i.e. the parietooccipital junction, frontal and temporal lobe, caudate nucleus and thalamus and the pre- and postcentral cortex.

A limitation of the study is the classification of amyloid-negative patients into the diagnostic categories FTD or severe psychiatric disorder, suspected 4R-tauopathy, and SNAP. SNAP does not represent a standalone clinical diagnosis but is a biomarker-based concept that is independent of the given level of cognitive impairment (Jack et al., 2016). It is a heterogeneous condition with several underlying pathomechanisms, such as cerebrovascular disease, argyrophilic grain disease, limbic-predominant age-related TDP-43 encephalopathy, or alpha-synucleinopathies potentially (Rizzi and Balthazar, 2021). Nevertheless, SNAP is a concept that is frequently encountered in clinical practice. As patients with SNAP exhibit a specific risk profile regarding the development of cognitive deficits, the concept is clinically relevant for counseling affected individuals. Therefore, we decided to include SNAP as a relevant diagnosis in our analysis.

Furthermore, FTD was pooled with severe psychiatric disorders in our analysis. While primary psychiatric disorder patients can exhibit hypometabolism that is most evident in the frontal lobes, the extent is typically less pronounced compared to FTD patients (Bystad et al., 2024, Lee et al., 2010, Ducharme et al., 2015). Due to this overlapping pattern of neurodegeneration, we decided to pool FTD and severe psychiatric disorder patients in our analysis. However, the fact that patients with primary psychiatric disorders occurred both in the A-N- and the A-N+ groups suggests that early-phase β-amyloid-PET is not well suited for accurately identifying these patients.

Moreover, the assumption that each clinical diagnosis corresponds to a specific perfusion pattern oversimplifies the heterogeneity of the dementia syndromes under investigation. While the distribution of neuronal injury assessed by early-phase β-amyloid-PET correlates with the clinical phenotype, it does not necessarily reflect the underlying pathogenic processes. As a result, the final clinical diagnosis may not always align with the detected perfusion pattern. Additionally, perfusion patterns may change over the course of the disease. Therefore, the pattern at the time of the β-amyloid-PET may differ from that at the time of the final clinical diagnosis. This temporal discrepancy could impact the classification and interpretation of perfusion patterns.

Another limitation that needs to be considered is the lack of histopathological validation. However, the absence of neuropathological validation poses a common challenge in research on rare neurodegenerative diseases. Consequently, there is a risk that some cases had a mismatch of clinical diagnosis and underlying pathology. However, a tight clinical follow-up after the PET scan (15.2 ± 17.6 months, min 0 months, max. 66 months) reassured us of the accuracy of the clinical diagnosis.

Also, it is important to note that z-scores for the early-phase β-amyloid-PET were obtained in comparison to normal [^18^F]FDG-PET data, rather than normal early-phase β-amyloid-PET data. This methodological choice was made to keep the analysis as close as possible to clinical practice, given the absence of early-phsae β-amyloid-PET reference cohorts in current databases. Nevertheless, in our small subpopulation of 15 A-N + patients who underwent both early-phase β-amyloid-PET and [^18^F]FDG-PET, regional mean z-scores demonstrated a strong spatial association across the 47 cerebral regions investigated (Spearman’s ρ = 0.733, Pearson’s r = 0.786; non-shared variance 46% and 38%, respectively), comparable to previous studies (Myoraku et al., 2022). However, given the small sample size, these results should be interpreted with caution. Overall these findings suggest that early-phase β-amyloid-PET reflects perfusion patterns similar to glucose metabolism, while still showing region-wise dissociations, indicating that perfusion and metabolism are related but not fully interchangeable, which might have influenced the results of our study.

Despite these limitations, our data reveal an additive value of early-phase β-amyloid-PET in the diagnostic work-up of amyloid-negative patients. Its ability to assess two biomarker categories in a single scan may eliminate the need for additional diagnostic procedures like [^18^F]FDG-PET, thus reducing costs, time and radiation exposure. While visual assessment of early-phase β-amyloid-PET data already provides substantial diagnostic accuracy, a data-driven analysis approach could offer additional assurance in cases of uncertainty.

## Authors contribution

All authors contributed were involved in data collection. Data analysis was performed by Sonja Schönecker. The first draft of the manuscript was written by Sebastian Eckenweber and Sonja Schönecker and all authors commented on previous versions of the manuscript. All authors read and approved the final manuscript.

## CRediT authorship contribution statement

**Sebastian Eckenweber:** Writing – original draft, Data curation, Conceptualization. **Friederike Völter:** Writing – review & editing, Data curation. **Nicolai Franzmeier:** Writing – review & editing, Visualization, Formal analysis. **Carla Palleis:** Writing – review & editing, Data curation. **Olivia Wagemann:** Writing – review & editing, Data curation. **Endy Weidinger:** Writing – review & editing, Data curation. **Sabrina Katzdobler:** Writing – review & editing, Data curation. **Elisabeth Wlasich:** Writing – review & editing, Data curation. **Katja Sandkühler:** Writing – review & editing, Data curation. **Guido Böning:** Writing – review & editing, Data curation. **Johannes Gnörich:** Writing – review & editing, Data curation. **Maximilian Scheifele:** Writing – review & editing, Data curation. **Florian Eckenweber:** Writing – review & editing, Data curation. **Daniel Janowitz:** Writing – review & editing, Data curation. **Carolin Kurz:** Writing – review & editing, Data curation. **Robert Perneczky:** Writing – review & editing, Data curation. **Katharina Bürger:** Writing – review & editing, Data curation. **Adrian Danek:** Writing – review & editing, Data curation. **Günter Höglinger:** Writing – review & editing, Data curation. **Johannes Levin:** Writing – review & editing, Data curation. **Matthias Brendel:** Writing – review & editing, Methodology, Data curation, Conceptualization. **Sonja Schönecker:** Writing – original draft, Visualization, Methodology, Formal analysis, Data curation, Conceptualization.

## 2.1 Ethics approval and consent to participate

The study was performed according to the Declaration of Helsinki (1991) and was approved by the local ethics committee (Medical Faculty, Ludwig-Maximilians-Universität München, Munich, Germany, 17–569). All participants provided written informed consent.

## Funding

The Friedrich-Baur-Stiftung and the “FöFoLe” Program of the Faculty of Medicine of the Ludwig-Maximilians-Universität München have supported the work of S.S. The Lüneburg Heritage and Friedrich-Baur-Stiftung have supported the work of C.P. This work was funded by the 10.13039/501100001659Deutsche Forschungsgemeinschaft (DFG, German Research Foundation) under Germany’s Excellence Strategy within the framework of the Munich Cluster for Systems Neurology (EXC 2145 SyNergy – ID 390857198).

## Declaration of competing interest

The authors declare that they have no known competing financial interests or personal relationships that could have appeared to influence the work reported in this paper.

## Data Availability

Data will be made available on request.
