# Supplementary material for: Additive value of early-phase β-Amyloid-PET for the differential diagnosis of non-Alzheimer’s disease dementia
Source: Neuroimage Clin. 2026 Feb 9;49:103963. doi: 10.1016/j.nicl.2026.103963 (PMC12925280; doi:10.1016/j.nicl.2026.103963)
Supplement: Supplementary Data 1 [file mmc1.docx]

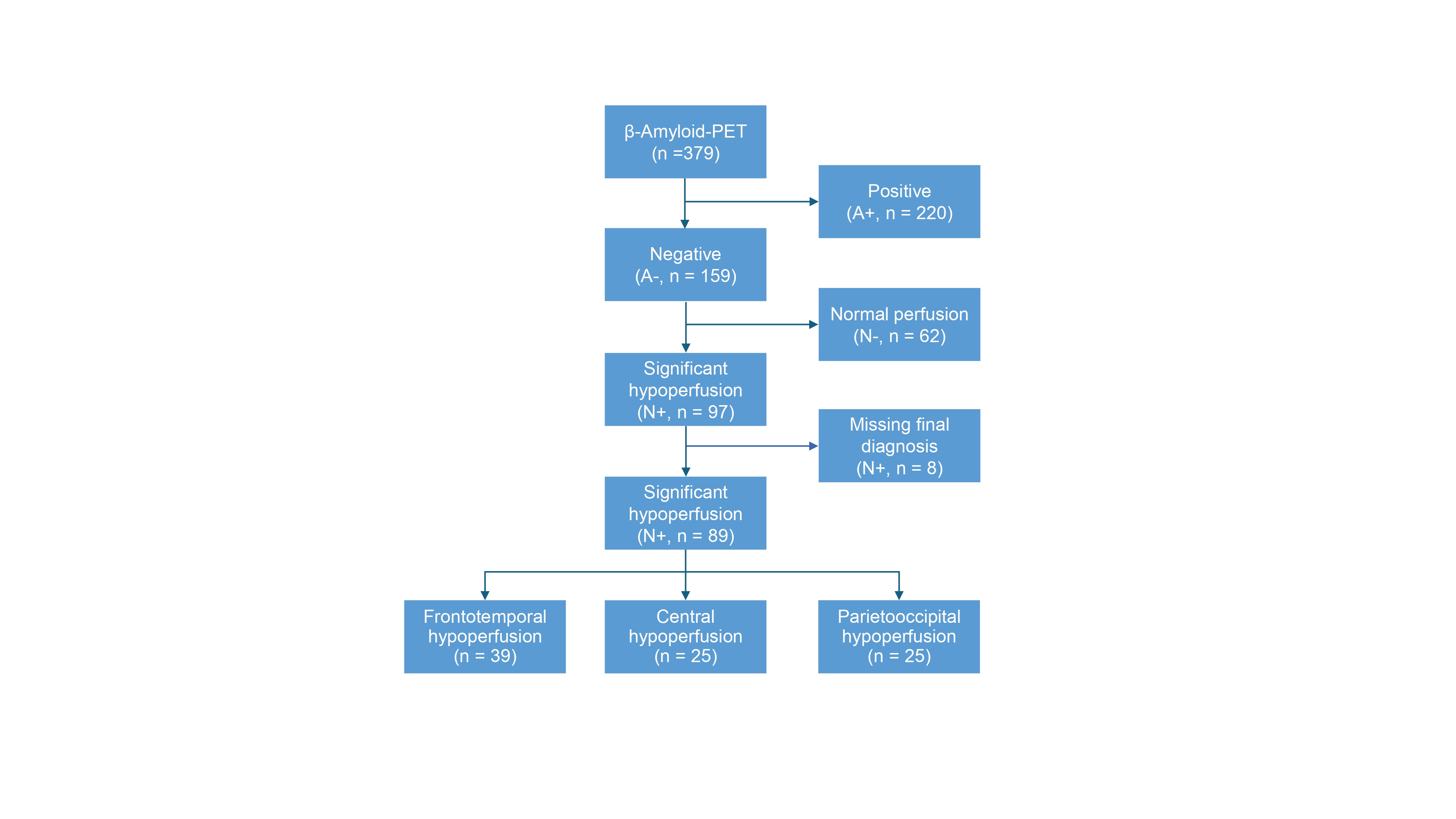


**Supplementary Fig. 1** Study flowchart. Patients whose late-phase images were rated as A- on visual inspection were considered as primary sample. The analysis focused on the subset of patients additionally being rated as N+.


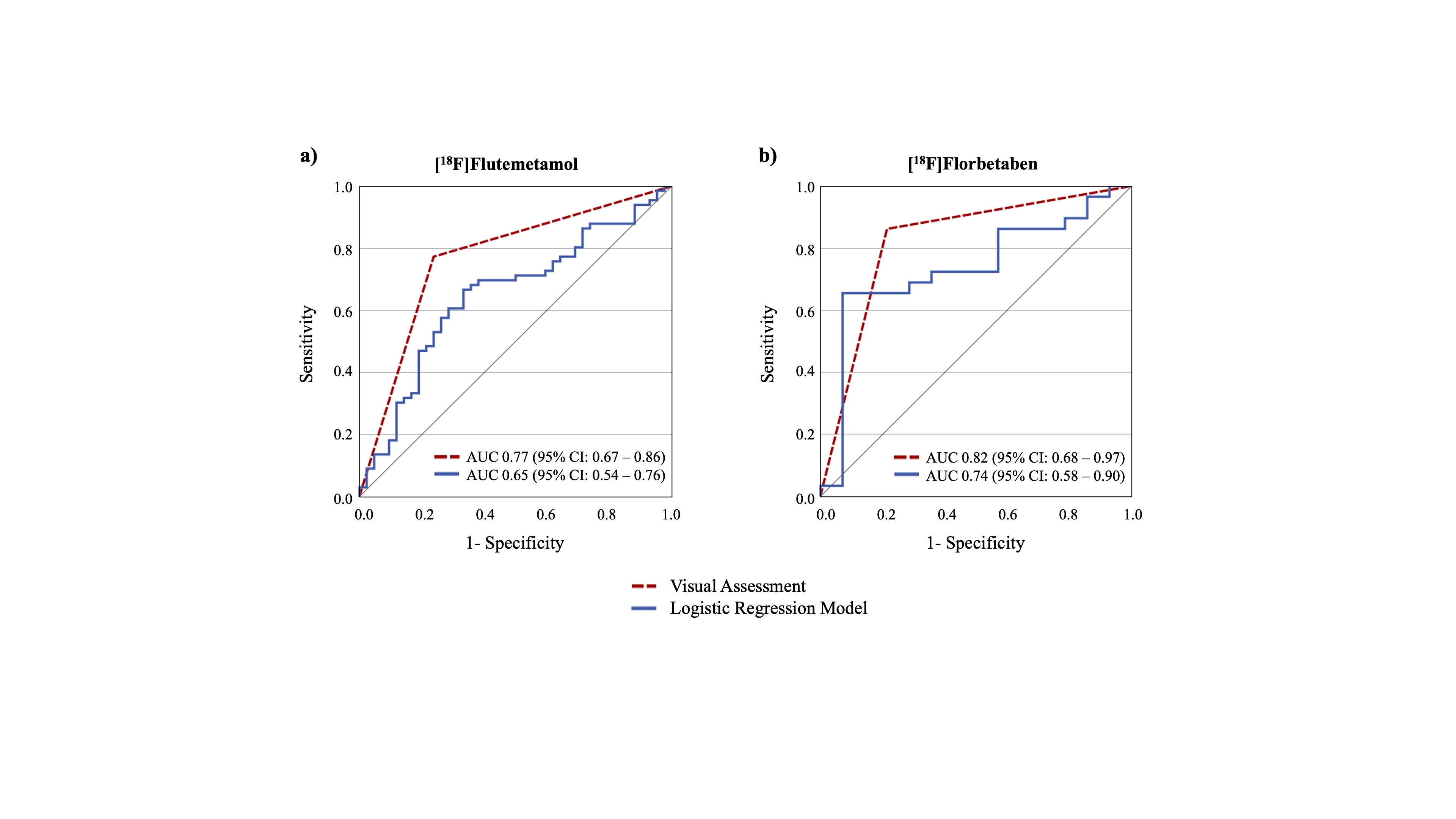


**Supplementary Fig. 2** Receiver operating characteristic (ROC) curves for the visual neurodegeneration assessment of early-phase β-amyloid-PET images versus logistic regression models, analyzed separately by tracer. (a) prediction of neurodegeneration status using [^18^F]flutemetamol, (b) prediction of neurodegeneration status using [^18^F]florbetaben. The black diagonal line is the ROC curve reference line. 95% CI, 95% confidence interval; AUC, area under the curve.


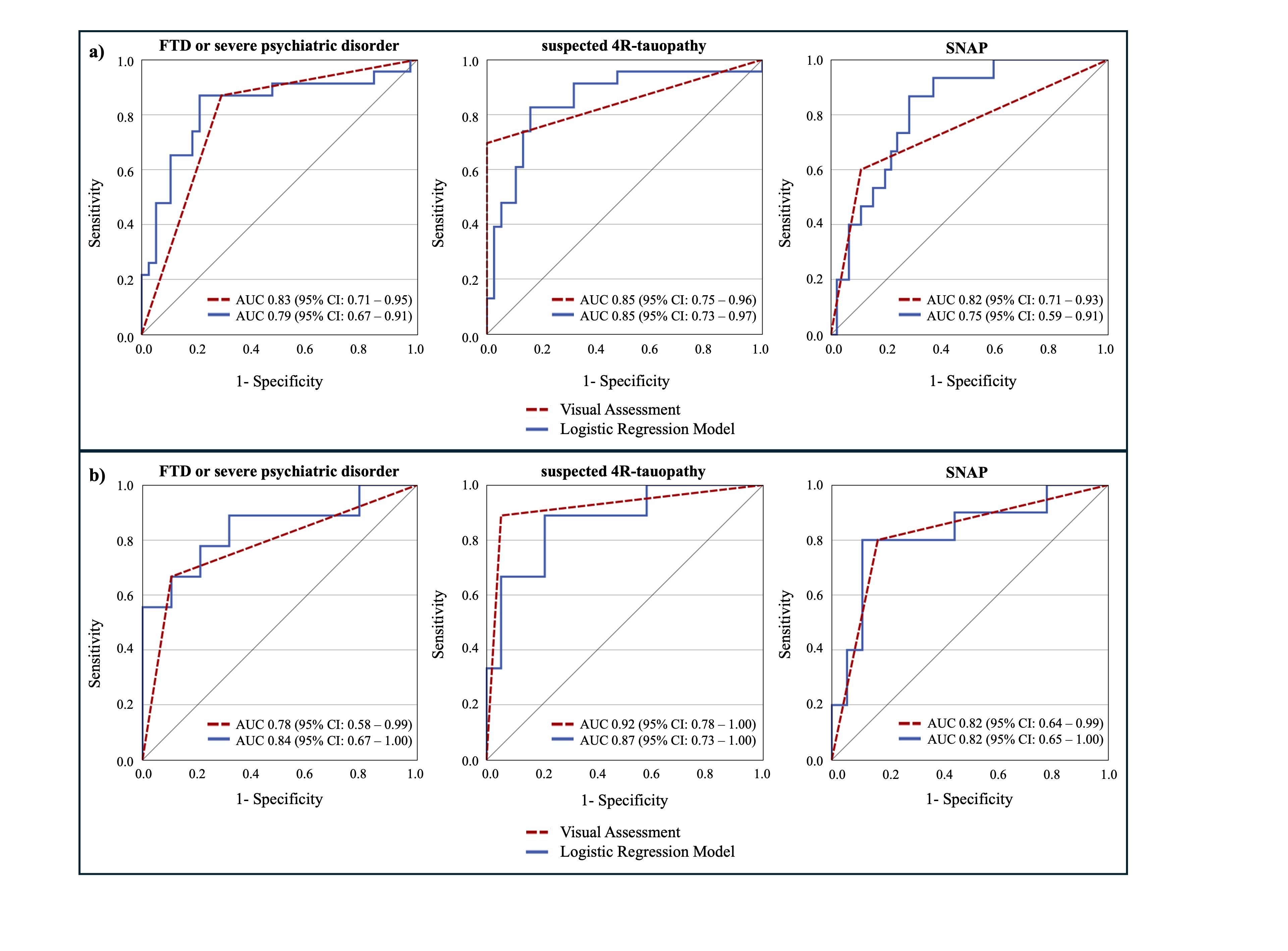


**Supplementary Fig. 3** Receiver operating characteristic (ROC) curves for the visual assessment of the final clinical diagnosis versus logistic regression models, analyzed separately by tracer. (a) prediction of the fincal clinical diagnosis, i.e. FTD or severe psychiatric disorder, suspected 4R-tauopathy and SNAP using [^18^F]flutemetamol, (b) prediction of the final clinical diagnosis using [^18^F]florbetaben. The black diagonal line is the ROC curve reference line. 95% CI, 95% confidence interval; AUC, area under the curve.


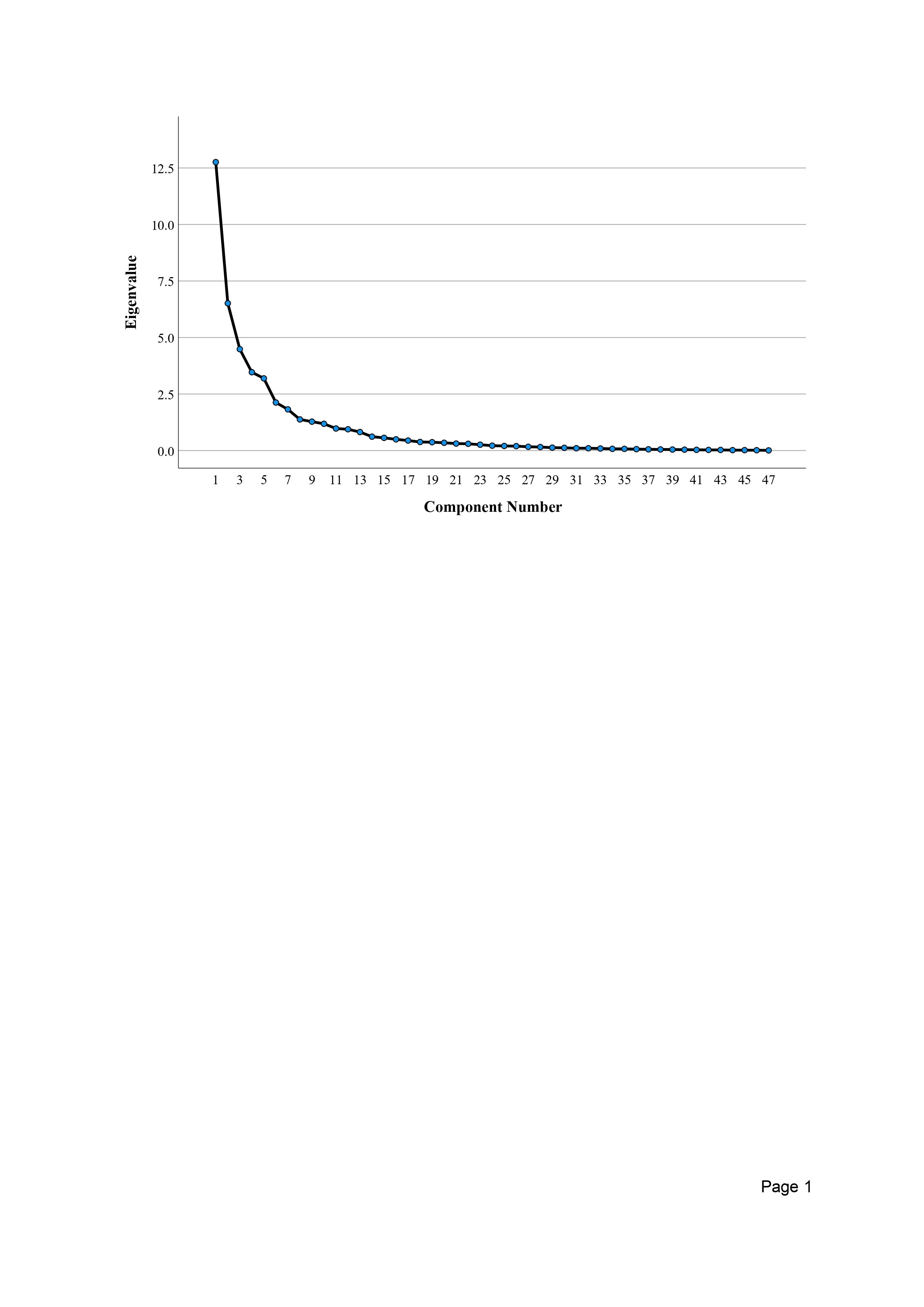


**Supplementary Fig. 4** Scree plot displaying the distribution of component numbers by their eigenvalues


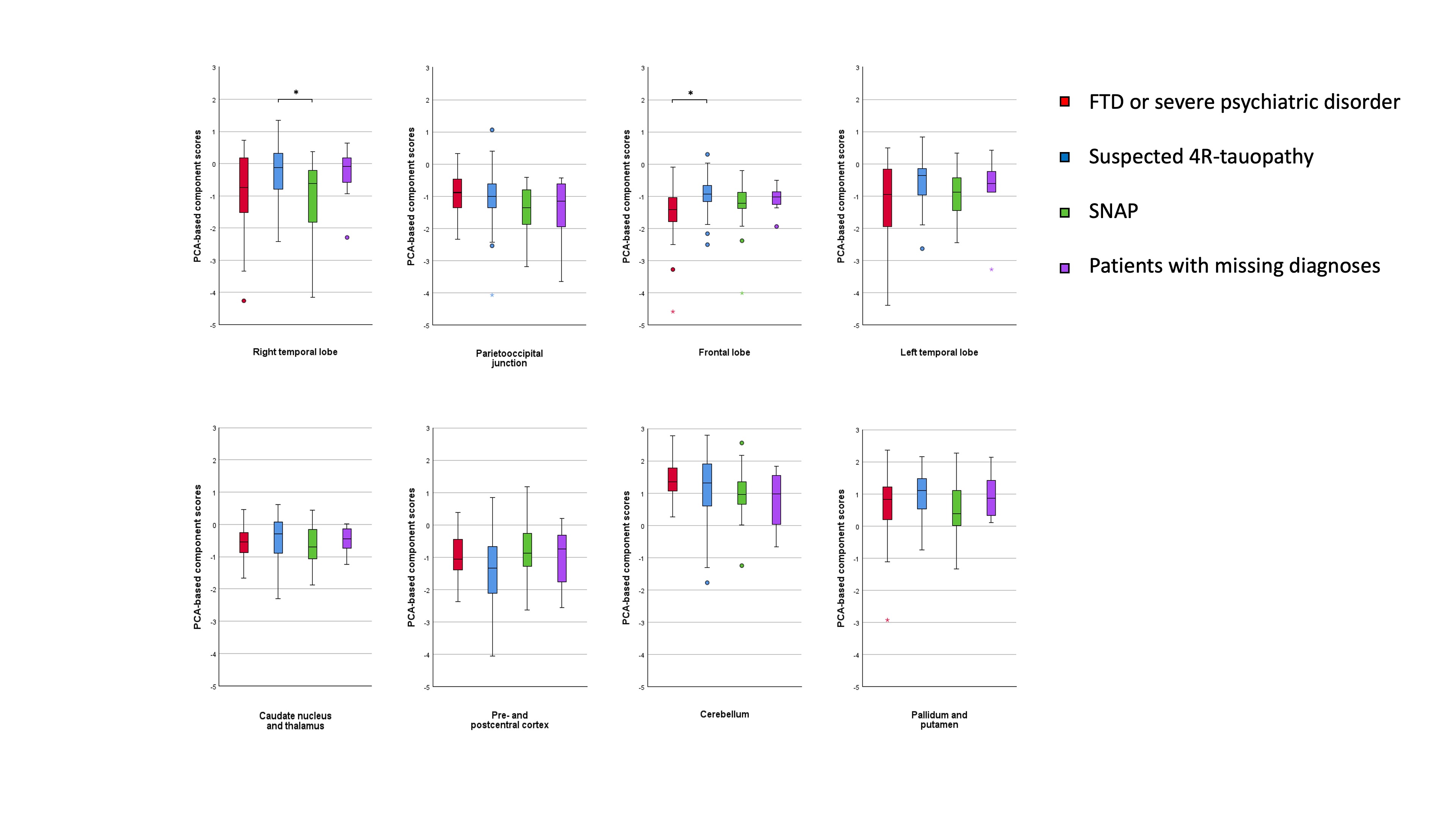


**Supplementary Fig. 5** Comparison of component scores between groups, including the 8 A-N+ patients excluded from the main analysis due to missing final clinical diagnosis. Component scores were calculated from the factor loading weighted variables of each of the components identified by applying principal component analysis on 47 cerebral regions. * indicates significant differences


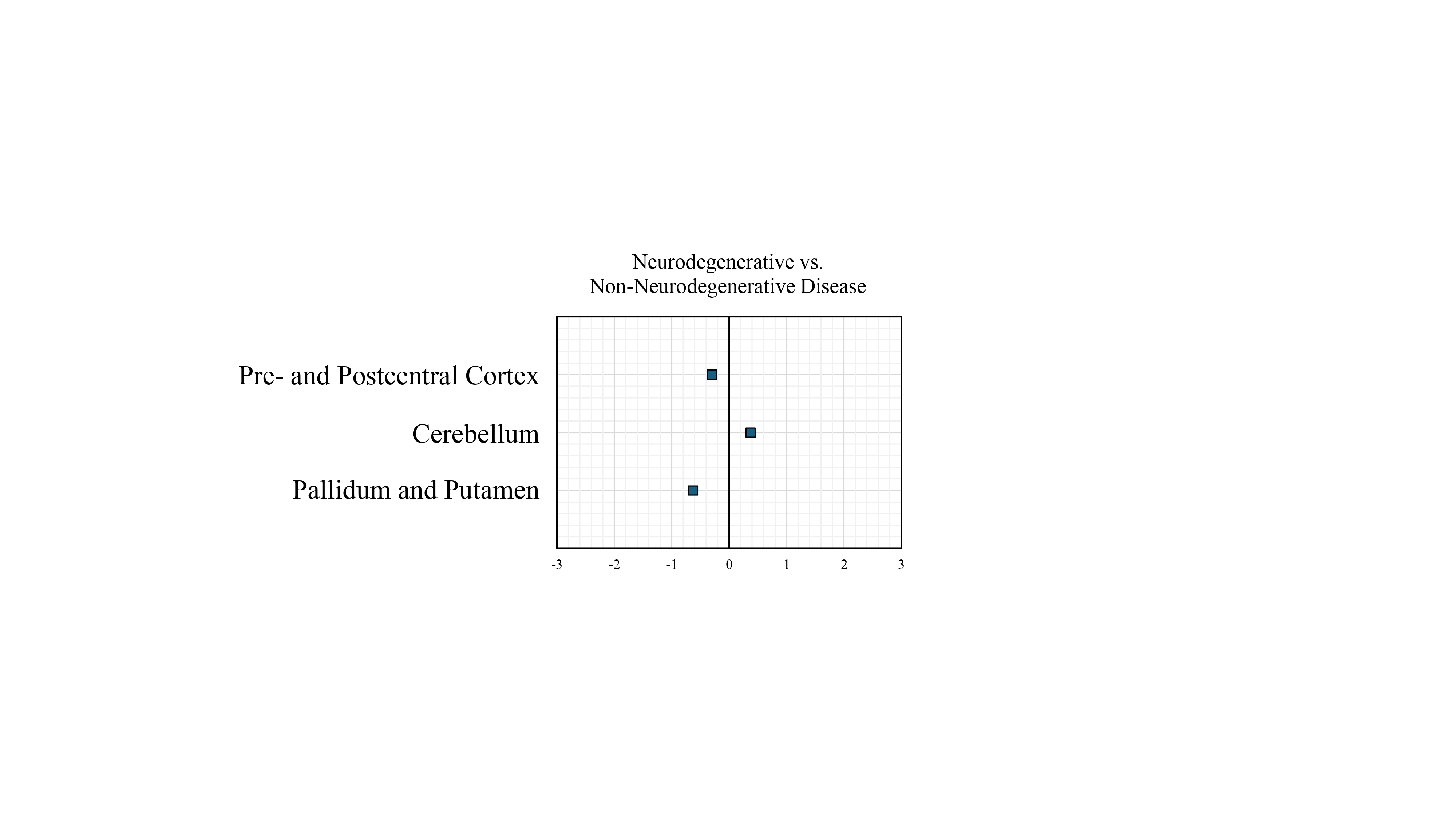


**Supplementary Fig. 6** Binary logistic regression coefficients. Coefficients for the 3 cerebral regions enclosed in the binary logistic regression model. A negative coefficient means that lower values (i.e. more hypoperfusion) on that variable increases the estimated probability of a neurodegenerative disease holding all other variables constant.


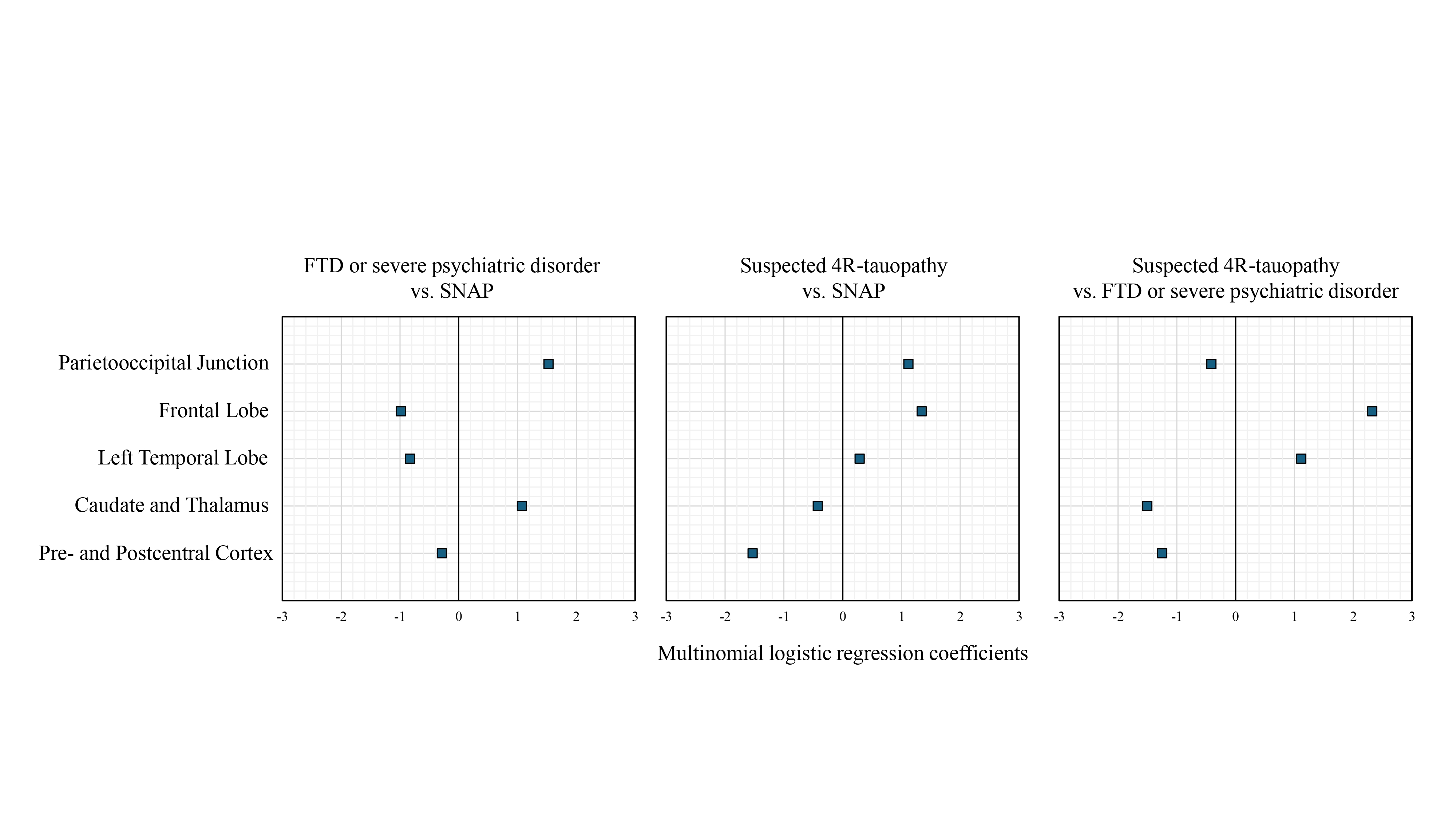


**Supplementary Fig. 7** Multinomial logistic regression coefficients. Coefficients for the 5 cerebral regions enclosed in the multinomial logistic regression model for the FTD or severe psychiatric disorder, suspected 4R-tauopathy and SNAP group against the respective reference group. A negative coefficient means that lower values (i.e. more hypoperfusion) on that variable increase the estimated probability of being in that group holding all other variables constant.


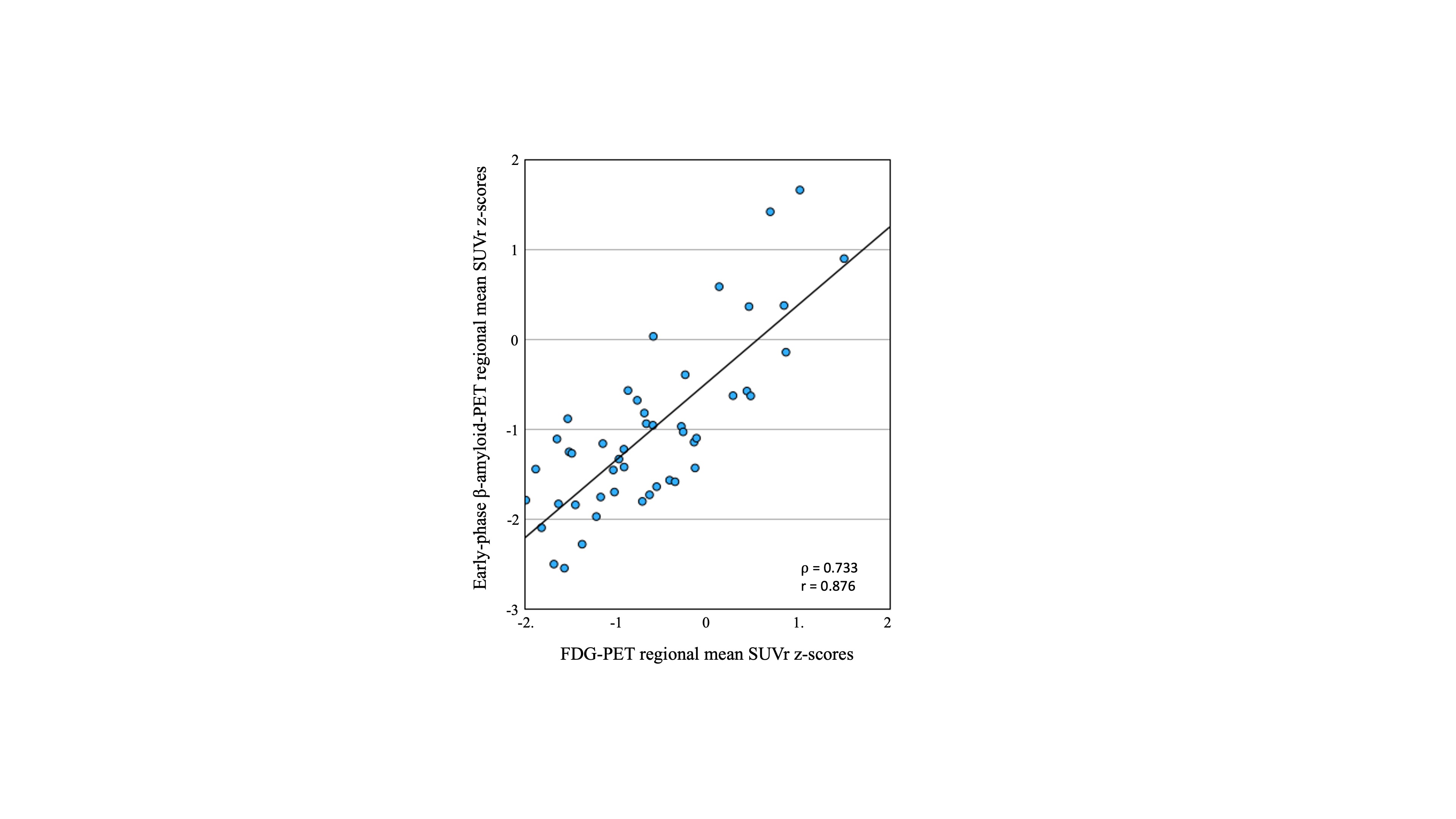


**Supplementary Fig. 8** Scatterplot of regional mean SUVr z-scores between early-phase β-amyloid-PET and FDG-PET across 47 cerebral regions in 15 A-N+ patients. Each point represents the regional mean SUVr z-score. Spearman’s rank correlation coefficient (ρ) is shown as the primary measure of association, while Pearson’s correlation coefficient (r) is provided for comparison. A linear regression line based on Pearson’s r is shown for illustration purposes.
